# A randomized, double-blind, placebo-controlled trial of olanzapine versus placebo plus ondansetron and dexamethasone for antiemetic prophylaxis in patients receiving oxaliplatin-, irinotecan-, or carboplatin-based chemotherapy

**DOI:** 10.1007/s00520-026-10490-8

**Published:** 2026-02-24

**Authors:** Warangkana Harikul, Akarin Nimmannit, Apirom Laocharoenkeat, Pochamana Phisalprapa, Chayanis Kositamongkol, Phurita Thongkijpreecha, Suthinee Ithimakin

**Affiliations:** 1 Division of Medical Oncology, Department of Medicine, Faculty of Medicine Siriraj Hospital, Mahidol University, Bangkok, Thailand; 2 Department of Research, Faculty of Medicine Siriraj Hospital, Mahidol University, Bangkok, Thailand; 3 Pharmacy Department, Siriraj Hospital, Bangkok, Thailand; 4 Division of Ambulatory Medicine, Department of Medicine, Faculty of Medicine Siriraj Hospital, Mahidol University, Bangkok, Thailand

**Keywords:** Olanzapine, Moderately emetogenic chemotherapy, Oxaliplatin, Irinotecan, Carboplatin

## Abstract

**Purpose:**

A two-drug regimen of palonosetron and dexamethasone is standard for moderately emetogenic chemotherapy (MEC), including oxaliplatin and irinotecan. Current guidelines recommend adding an NK1 receptor antagonist for carboplatin-based or MEC in patients with high-risk features. Given the comparable efficacy of olanzapine, this study evaluated the effectiveness of low-dose olanzapine (OLN, 5 mg) combined with ondansetron and dexamethasone in preventing chemotherapy-induced nausea and vomiting (CINV).

**Methods:**

In this double-blind, randomized controlled trial, patients initiating oxaliplatin-, carboplatin-, or irinotecan-based chemotherapy were randomized 1:1 to receive OLN or placebo on Days 1–4, with ondansetron and dexamethasone. Randomization was stratified by chemotherapy type and high-risk factors (female aged < 50 years). The primary endpoint was total protection (mild/no nausea, no vomiting, and no rescue therapy) within 120 h post-chemotherapy. Secondary endpoints included total control, complete response, nausea/vomiting severity, rescue use, adverse events, and patient satisfaction.

**Results:**

Among 139 evaluable patients, 69 received OLN and 70 received a placebo. Total protection was achieved in 71.0% of OLN patients versus 55.7% with placebo (*p* = 0.06). Total control was significantly higher with OLN (62.3% vs. 38.6%, *p* = 0.005). Delayed nausea (grade ≥ 2) occurred less frequently with OLN (13.0% vs. 30.0%, *p* = 0.015). Complete response and rescue use did not differ between groups. Somnolence rates were similar, but anorexia was less familiar with OLN. Notably, 95.6% of OLN patients preferred to continue the same regimen, compared with 72.9% of placebo recipients (*p* = 0.001).

**Conclusion:**

Olanzapine (5 mg) combined with ondansetron and dexamethasone was associated with a moderate improvement in total protection and significant improvements in no-nausea and total control rates.

**Supplementary Information:**

The online version contains supplementary material available at 10.1007/s00520-026-10490-8.

## Introduction

Chemotherapy-induced nausea and vomiting (CINV**)** remain one of the most common and distressing adverse effects of cancer treatment. Beyond patient discomfort, CINV can result in serious complications such as dehydration, electrolyte disturbances, malnutrition, fatigue, and progressive functional decline. These consequences markedly impair quality of life and may compromise adherence to subsequent chemotherapy cycles, thereby undermining the overall effectiveness of cancer therapy. Although newer antiemetic agents have been integrated into triplet or quadruplet regimens for highly emetogenic chemotherapy (HEC), their consistent use in prophylaxis for moderately emetogenic chemotherapy (MEC) remains limited.

Without adequate prophylaxis, CINV develops in approximately 30–90% of patients within the first 24 h after administration of MEC [1]. Agents commonly classified as MEC in routine practice include oxaliplatin and irinotecan, both widely employed in gastrointestinal cancers. Carboplatin, classified as MEC by the Multinational Association of Supportive Care in Cancer/European Society for Medical Oncology (MASCC/ESMO) and the American Society of Clinical Oncology (ASCO) guidelines [2], is considered to lie at the upper end of the moderate emetogenic spectrum. However, given its substantial emetogenic potential, the National Comprehensive Cancer Network (NCCN) recommends that carboplatin be administered at an area under the concentration-time curve (AUC) of ≥ 4 to be categorized as HEC.

While evidence for neurokinin-1 receptor antagonists (NK1-RAs) in MEC continues to grow, data on the antiemetic benefit of olanzapine in this setting remain limited. Retrospective analyses of olanzapine in MEC regimens containing oxaliplatin or irinotecan suggest improved emetic control and better quality of life compared with non-olanzapine dual regimens [3]. A randomized controlled trial evaluating olanzapine 10 mg with palonosetron and dexamethasone versus placebo in MEC reported similar complete response rates across groups. However, patients receiving olanzapine experienced superior nausea control and required fewer rescue medications [4]. More recently, a large randomized controlled trial [5] demonstrated enhanced CINV control when olanzapine 10 mg was added to a three-drug regimen of aprepitant, palonosetron, and dexamethasone, aligning with prior trials conducted in both HEC and MEC populations [4, 6].

According to the 2023 MASCC/ESMO guidelines, recommendations for MEC remain variable. The standard approach continues to be a two-drug combination of a 5-HT3 receptor antagonist (5-HT3 RA) and dexamethasone for both acute and delayed CINV prophylaxis. Routine use of a three-drug antiemetic regimen is not generally advised. However, for high-risk subgroups—such as women under 50 years—the addition of an NK1-RA may be appropriate. Given that olanzapine has shown comparable efficacy to NK1-RAs in HEC settings and is more cost-effective, it is increasingly considered as a third agent with a 5-HT3 RA plus dexamethasone in MEC prophylaxis [7]. Nevertheless, phase III evidence supporting this strategy in the general MEC population remains sparse.

In recent years, interest has increased in assessing olanzapine’s role in MEC, particularly among patients at elevated risk of CINV. A retrospective study reported lower CINV incidence when olanzapine was added to oxaliplatin- or irinotecan-based regimens in high-risk individuals, such as women under 55 years without a history of alcohol use [3]. A prospective trial involving female patients with gastrointestinal cancers receiving FOLFOX or FOLFIRI showed significantly improved acute-phase nausea control in the olanzapine arm. However, no significant differences were observed during the delayed phase [8].

For carboplatin-based regimens, tailored antiemetic recommendations exist because of their relatively high emetogenic potential. A three-drug regimen with palonosetron, dexamethasone, and an NK1-RA is generally advised [2]. Several studies have evaluated olanzapine as an alternative to NK1-RAs in this context and demonstrated meaningful improvements in nausea and vomiting control [9, 10]. However, other trials—particularly those examining carboplatin/paclitaxel combinations—found no added benefit from olanzapine compared with standard doublet therapy [11] or when incorporated into triplet regimens that already included an NK1-RA [12].

A review of the current literature suggests that olanzapine may improve CINV prevention, particularly by increasing the likelihood of patients remaining nausea-free without requiring rescue medication. However, robust phase III evidence supporting its use as a third agent in MEC regimens with carboplatin, oxaliplatin, or irinotecan is still limited. Notably, most studies reporting benefit have employed palonosetron as the 5-HT3 RA [4, 8, 12], whereas in real-world practice in Thailand, ondansetron is more commonly prescribed and is the only reimbursable 5-HT3 RA [13].

Given the absence of clinical data on olanzapine combined with ondansetron and dexamethasone for MEC prophylaxis, this study was designed to evaluate the efficacy of olanzapine 5 mg versus placebo, in combination with ondansetron and dexamethasone, among patients receiving oxaliplatin-, irinotecan-, or carboplatin-based chemotherapy.

## Patients and methods

### Study design

This randomized, double-blind, placebo-controlled trial evaluated the efficacy of olanzapine 5 mg (OLN5), combined with ondansetron and dexamethasone, for preventing CINV in patients receiving MEC. Eligible participants were treated with carboplatin, irinotecan, or oxaliplatin at Siriraj Hospital between December 2024 and July 2025. Randomization was performed in a 1:1 ratio using a computer-generated sequence with variable mixed block randomization, assigning patients to either OLN5 or placebo in addition to standard prophylaxis with ondansetron and dexamethasone. Stratification was based on chemotherapy regimen (e.g., carboplatin AUC ≥ 5, oxaliplatin-based, or irinotecan-based) and presence of high-risk features (female sex aged < 50 years). Allocation concealment was used with sequentially numbered, opaque, sealed envelopes prepared by an investigator. Randomization sequence and envelope preparation were conducted by different investigators. Participant enrollment was conducted by a separate investigator, who opened the envelopes sequentially only after an eligible participant had been enrolled. Both OLN5 and placebo were encapsulated in identical capsules by a blinded pharmacist and dispensed by another pharmacist who was also blinded. All participants were monitored for 5 days after chemotherapy. The study protocol was approved by the Siriraj Institutional Review Board (Certificate of Approval No. Si849/2024) and supported by a research grant from the Faculty of Medicine, Siriraj Hospital, Mahidol University (Grant Number IO R016835007). Written informed consent was obtained from all participants.

### Patients

Eligible patients were ≥ 18 years old, had histologically confirmed solid tumors, and were scheduled to receive their first MEC cycle containing carboplatin (AUC ≥ 5), oxaliplatin, or irinotecan. Patients were excluded if they had received other highly or MEC within the same cycle as part of a combination regimen or as multi-day therapy or if they had experienced nausea or vomiting within 24 h before treatment.

Exclusion criteria included pregnancy, untreated gastrointestinal obstruction, abdominal or pelvic radiotherapy within 1 week of enrollment, uncontrolled brain metastases, significant cardiovascular disease, history of allergy or serious adverse reactions to any study drug (ondansetron, olanzapine, or dexamethasone), or current olanzapine use. Adequate renal and hepatic function was required, defined as total bilirubin ≤ 2 mg/dL and creatinine clearance ≥ 30 mL/min.

### Treatments

All patients received a single intravenous dose of ondansetron (8 mg) and dexamethasone (12 mg) 30 min before chemotherapy. Oral ondansetron (8 mg) was administered twice daily on Days 2–4. Patients in the experimental group received OLN5 orally prior to chemotherapy administration on Day 1 and once daily at bedtime from Day 2 to Day 4, while those in the control group received a matching placebo on the same schedule. Rescue antiemetics were permitted at the discretion of the treating physician in routine clinical practice. Preemptive prescription was not provided. Unblinding was permitted at any time upon the treating physician’s request if clinically necessary to guide further antiemetic management.

### Assessment

Patients completed a daily diary for 120 h (Day 1 through Day 5 post-chemotherapy) documenting vomiting episodes, nausea, and sedation severity (measured on a 0–10 visual analog scale [VAS]), rescue medication use, satisfaction, and preferred antiemetic choice for subsequent cycles, along with any adverse events. During the observation period, participants were managed in inpatient or outpatient settings according to the treating physician’s discretion, in accordance with routine clinical practice. On Days 2 and 5, a blinded assessor contacted participants in both groups by telephone or through the LINE Official Account (per patient preference) to provide reminders and collect interim symptom data. Patients returned completed diaries and any unused medication at their next scheduled visit.

CINV severity was graded using the Common Terminology Criteria for Adverse Events (CTCAE), version 3.0. Nausea and sedation were assessed with the VAS, ranging from 0 (none) to 10 (most severe). CINV outcomes were classified as acute (0–24 h), delayed (24–120 h), or overall (0–120 h). Because of the sedative potential of olanzapine, sedation was specifically measured with the VAS and recorded daily. Participants were instructed to record the worst symptoms experienced during the preceding 24 h. Diary entries were completed 24 h after chemotherapy administration (D1) and then at 24-h intervals through D5.

### Outcomes

The primary endpoint was total protection (TP), defined as no vomiting, no rescue medication use, and a VAS nausea score < 25 mm. Secondary endpoints included no-nausea rate, CINV severity, VAS nausea scores, complete response (CR), total control (TC), rescue medication use, and adverse events. TC was defined as a complete absence of nausea (VAS = 0) with no vomiting and no rescue therapy. CR was defined as no vomiting and no rescue therapy, irrespective of nausea. In cases of incomplete diary data, the worst available symptoms were reported and used for analysis.

### Statistical analysis

Descriptive statistics summarized baseline characteristics. Categorical variables were compared with Pearson’s chi-square test, and continuous variables (e.g., VAS scores) were compared with independent *t*-tests. Analyses were performed on a per-protocol basis using SPSS, version 20 (IBM Corp., Armonk, NY, USA).

Based on prior data showing a 40% TP rate with palonosetron and dexamethasone [3], we hypothesized that adding OLN5 to ondansetron/dexamethasone would increase the TP rate to 65%. To detect this difference with 80% power at a two-sided alpha of 0.05, 62 patients per group were required. Allowing for 10% attrition, the target enrollment was 70 per arm, for a total of 140 participants.

In addition, a cost-effectiveness analysis was performed to estimate the medication cost required to achieve one additional case of TP, TC, CR, or prevention of nausea. Full methodological details are available in the Supplementary Data.

## Results

### Patient characteristics

**Fig. 1:**
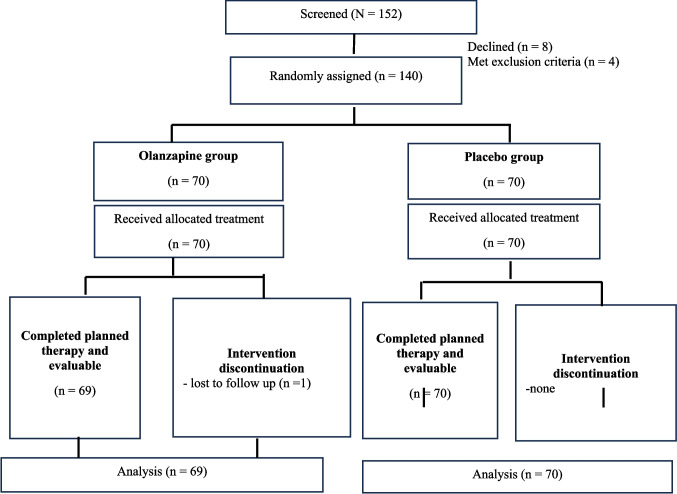
Schematic representation of the study. n, number of patients

A total of 140 patients were enrolled, 139 were included in the final analysis (Fig. 1). Sixty-nine patients were randomized to OLN5 and 70 to placebo. Baseline characteristics were comparable between groups, including known CINV risk factors such as younger age, female sex, history of motion sickness, and alcohol use (Table 1). The median age was 63 years (range, 32–82). Female distribution was similar, with 33 (41.7%) in the placebo group and 28 (40.6%) in the OLN5 group. High-risk CINV, defined as female patients younger than 50, was seen in eight patients—five on placebo and three on OLN5. Most patients had excellent performance status. The most common chemotherapy regimens were oxaliplatin-based, followed by carboplatin-based and irinotecan-based.

**Table 1:** Patient baseline characteristics

Characteristics	Placebo (*N* = 70)	Olanzapine (*N* = 69)
Age, median, (range), years	62.74 (34.8, 78.05)	63.43 (32.13, 82.11)
Gender, n (%)		
Female	33 (41.7)	28 (40.6)
Male	37 (52.9)	41 (59.4)
Female < 50 years, n (%)	5 (7.1)	3 (4.3)
Primary cancer, n (%)		
Lung	12 (17.1)	13 (18.8)
Gastrointestinal tract	50 (71.5)	48 (69.7)
Others	8 (11.4)	8 (11.5)
Performance status, n (%)		
0–1	65 (92.9)	64 (92.7)
≥ 2	5 (7.1)	5 (7.2)
Chemotherapy, n (%)		
Carboplatin	21 (30)	20 (29)
Oxaliplatin	45 (64.3)	45 (65.2)
Irinotecan	4 (5.7)	4 (5.8)
Line of treatment, n (%)		
1st	61 (87.1)	58 (84.1)
2nd	9 (12.9)	11 (15.9)
Alcohol use, n (%)	10 (15.6)	9 (13.6)
History of motion sickness, n (%)	3 (5.1)	4 (6.8)
History of pregnancy sickness, n (%)	5 (7.1)	4 (6.8)

*N* total number of patients, *n* number of patients

### CINV efficacy

CINV outcomes are summarized in Table 2. A trend toward improved TP rate in the overall period was noted with OLN5 (71% vs. 55.7%, *p* = 0.061). Olanzapine significantly improved TC and no-nausea rates compared with placebo. In the overall phase, more OLN5 patients achieved TC—defined as no vomiting, no rescue medication, and a VAS nausea score of 0—than placebo (62.3% vs. 38.6%, *p* = 0.005). During the acute phase (0–24 h), the OLN5 group again showed higher TC rates (84.1% vs. 65.7%, *p* = 0.013). The no-nausea rate was also greater with OLN5 in the overall (65.2% vs. 38.6%, *p* = 0.002), acute (84.1% vs. 65.7%, *p* = 0.013), and delayed phases (65.2% vs. 42.9%, *p* = 0.008). However, the CR rate and rescue medication use did not differ significantly between groups.

**Table 2:** Proportion of patients achieving chemotherapy-induced nausea and vomiting (CINV) control

Endpoints	Placebo (*N* = 70)	Olanzapine (*N* = 69)	*p* value
	*n* (%)	*n* (%)	
Total protection rate			
Overall	39 (55.7)	49 (71)	0.061
Acute (0–24 h)	58 (82.9)	62 (89.9)	0.23
Delayed (24–120 h)	39 (55.7)	49 (71)	0.061
Total control rate			
Overall	27 (38.6)	43 (62.3)	0.005
Acute (0–24 h)	46 (65.7)	58 (84.1)	0.013
Delayed (24–120 h)	30 (42.9)	43 (62.3)	0.022
Complete response rate			
Overall	46 (65.7)	55 (79.7)	0.064
Acute (0–24 h)	70 (100)	69 (100)	1
Delayed (24–120 h)	46 (65.7)	55 (79.7)	0.064
No nausea			
Overall	27 (38.6)	45 (65.2)	0.002
Acute (0–24 h)	46 (65.7)	58 (84.1)	0.013
Delayed (24–120 h)	30 (42.9)	45 (65.2)	0.008
Rescue therapy			
Overall	3 (4.3)	1 (1.4)	0.317
Acute (0–24 h)	0	0	1
Delayed (24–120 h)	3 (4.3)	1 (1.4)	0.317

*h* hours, *N* total number of patients, *n* number of patients

The cost-effectiveness analysis showed that, compared with placebo, the incremental cost-effectiveness ratios for OLN5 were 633.21 Thai baht per additional case of TP, 407.97 baht per case of TC, 692.23 baht per CR, and 363.59 baht per non-nausea case (Table S1).

Across chemotherapy subgroups, OLN5 provided consistent clinical benefit. Improved TC rates were observed in patients receiving carboplatin-, oxaliplatin-, and irinotecan-based regimens (Table 3).

**Table 3:** Chemotherapy-induced nausea and vomiting (CINV) control by chemotherapy type during the overall period

Endpoints	Placebo	Olanzapine	*p* value
	*n* (%)	*n* (%)	
Carboplatin			
TC rate	8/21 (38.1)	14/20 (70)	0.041
TP rate	12/21 (57.1)	14/20 (70)	0.393
CR rate	15/21 (71.4)	15/20 (75)	0.796
Rescue therapy	1/21 (4.8)	0	0.323
Oxaliplatin			
TC rate	17/45 (37.8)	25/45 (55.6)	0.091
TP rate	24/45 (53.3)	31/45 (68.9)	0.13
CR rate	28/45 (62.2)	36/45 (80)	0.063
Rescue therapy	2/45 (4.4)	1/45 (2.2)	0.557
Irinotecan			
TC rate	2/4 (50)	4/4 (100)	0.102
TP rate	3/4 (75)	4/4 (100)	0.285
CR rate	3/4 (75)	4/4 (100)	0.285
Rescue therapy	0	0	-

*n* number of patients, *TC* total control, *TP* total protection, *CR* complete response

Table 4 summarizes CINV severity by CTCAE criteria. OLN5 patients experienced fewer nausea episodes than those on placebo during both the overall phase (47.8% vs. 78.6%, *p* < 0.001) and delayed phase (47.8% vs. 77.1%, *p* < 0.001). Moderate to severe (grade 2–4) nausea was also less frequent with OLN5 in the overall phase (15.9% vs. 31.4%, *p* = 0.032) and delayed phase (13.0% vs. 30.0%, *p* = 0.015). Despite these improvements, median VAS nausea scores were low in both groups and did not differ significantly. No acute-phase vomiting was reported in either group. In the delayed phase, vomiting was more common in the placebo (32.9% vs. 21.7%, *p* = 0.141), including grade > 2 (11.4% vs. 5.8%, *p* = 0.237), though differences were not statistically significant.

**Table 4:** Degree of chemotherapy-induced nausea and vomiting

Degree of CINV	Placebo (*N* = 70)	Olanzapine (*N* = 69)	*p* value
	*n* (%)	*n* (%)	
Overall			
Any nausea	55 (78.6)	33 (47.8)	< 0.001
Grades 2–4 nausea	22 (31.4)	11 (15.9)	0.032
Any vomiting	23 (32.9)	15 (21.7)	0.141
Grades 2–4	8 (11.4)	4 (5.8)	0.237
Acute (0–24 h)			
Any nausea	26 (37.1)	18 (26.1)	0.161
Grades 2–4 nausea	3 (4.3)	7 (10.3)	0.173
Any vomiting	0	0	0
Grades 2–4 vomiting	0	0	0
Delayed (24–120 h)			
Any nausea	54 (77.1)	33 (47.8)	< 0.001
Grades 2–4 nausea	21 (30)	9 (13)	0.015
Any vomiting	23 (32.9)	15 (21.7)	0.141
Grades 2–4 vomiting	8 (11.4)	4 (5.8)	0.237
VAS nausea, median (range)			
Acute (0–24 h)	1.07 (0, 8)	0.57 (0, 8)	0.1
Delay (24–120 h)	1.45 (0, 10)	2.19 (0, 9)	0.096

*h* hours, *N* total number of patients, *n* number of patients

### Adverse events

Most adverse events were attributed to chemotherapy rather than the study drug. Constipation was the most notable event in the OLN5 group, though its incidence did not differ significantly from placebo. No severe adverse events, including neurologic or cardiac complications, were observed. Interestingly, anorexia was significantly less frequent with OLN5 (26.1% vs. 47.1%, *p* = 0.01).

Although somnolence is a known effect of olanzapine, both incidence and VAS sedation scores were similar between groups. On Day 1, the OLN5 group showed slightly higher sedation scores than placebo (3.14 vs. 2.11, *p* = 0.05), suggesting a borderline increase immediately post-administration. No significant differences were seen from Days 2–5 (Table 5).

**Table 5:** Adverse events

Adverse events	Placebo (*N* = 70)	Olanzapine (*N* = 69)	*p* value
	*n* (%)	*n* (%)	
Constipation	20 (28.6)	28 (40.6)	0.137
Headache	8 (11.4)	6 (8.7)	0.592
Diarrhea	7 (10)	4 (5.8)	0.359
Anorexia	33 (47.1)	18 (26.1)	0.01
Hiccup	12 (17.1)	13 (18.8)	0.794
Fatigue	29 (41.4)	28 (40.6)	0.919
Abdominal bloating	2 (2.9)	6 (8.7)	0.139
Neurological	7 (10)	3 (4.3)	0.197
Somnolence	22 (31.4)	24 (38.4)	0.674
VAS sedation score, mean (range)			
Day 1	2.11 (0, 10)	3.14 (0, 10)	0.05
Day 2–5	2.43 (0, 8)	2.75 (0, 10)	0.48

*N* total number of patients, *n* number of patients

### Patients’ satisfaction

Overall satisfaction scores were high in both groups (rated 4–5 on a 5-point scale) and did not differ significantly (*p* = 0.273). However, more OLN5 patients preferred continuing the same antiemetic regimen in future cycles (95.6% vs. 72.9%, *p* = 0.001). Conversely, the expectation to escalate antiemetic therapy was more common in the placebo (22.9%) than in OLN5 (4.4%). No patient in the OLN5 group suggested a dose reduction due to adverse effects. Treatment adherence was excellent, with > 97% of participants completing the full regimen (Table 6).

**Table 6:** Patient satisfaction

Patients report outcomes	Placebo (N = 70)	Olanzapine (N = 69)	*p* value
	*n* (%)	*n* (%)	
Satisfaction score (range of 1–5)			
Low (score 1–2)	5 (7.1)	2 (2.9)	0.273
Moderate (score 3)	15 (21.4)	10 (14.5)	
High (score 4–5)	50 (71.4)	56 (81.2)	
Missing	0	1 (1.4)	
Preferred antiemetic in subsequent chemotherapy cycle			
Same regimen	51 (72.9)	65 (94.2)	0.001
Escalation due to CINV	16 (22.8)	3 (4.3)	
Reduced due to adverse events	3 (4.3)	0 (0)	
Missing	0	1 (1.4)	
Completion of assigned therapy			
Complete	68 (97.1)	68 (98.6)	0.568
Not complete	2 (2.9)	1 (1.4)	

*CINV* chemotherapy-induced nausea vomiting, *N* total number of patients, *n* number of patients

## Discussion

The role of adding a third agent—such as olanzapine or a neurokinin-1 receptor antagonist (NK1-RA)—to antiemetic regimens for patients receiving MEC remains debated. Guidelines suggest considering an NK1-RA for patients with high-emesis risk, whereas the 2025 NCCN guidelines go further, advocating routine triplet therapy with either an NK1-RA or olanzapine to optimize CINV control [7].

To our knowledge, this is the first randomized controlled trial to evaluate low-dose olanzapine added to ondansetron plus dexamethasone for CINV prevention in a general MEC population receiving high-dose carboplatin-, oxaliplatin-, or irinotecan-based chemotherapy. While prior studies demonstrated benefit in high-risk groups—particularly younger female patients with gastrointestinal cancers [3, 8]—our study aimed to assess OLN5 in a broader population, potentially supporting an expanded role in this setting.

Our results showed a trend toward improved TP and CR rates with OLN5, along with significantly higher TC and no-nausea rates. These findings highlight its efficacy in nausea prevention, particularly during the delayed phase of CINV [3, 4, 6, 8, 12, 13]. OLN5 reduced the proportion of patients reporting mild nausea, though rates of moderate to severe nausea, CR, and rescue medication use were similar. This suggests that while OLN5 may not substantially affect emesis in the overall population, it meaningfully improves nausea control—especially milder symptoms that may still impair quality of life. Although quality of life was not formally assessed, high patient satisfaction and preference for continuing the same regimen in subsequent cycles provide indirect evidence of clinical benefit. The safety profile of OLN5 was manageable, with no significant increase in somnolence severity. Notably, fewer patients in the OLN5 group reported anorexia compared with placebo.

Our results align with a prior randomized trial of olanzapine 10 mg combined with palonosetron and dexamethasone, particularly for nausea control and CR [4]. When TC is used as the primary endpoint, low-dose olanzapine added to a triplet regimen—including an NK1-RA, palonosetron, and dexamethasone—has also been shown to improve TC and quality of life [12, 14]. While our findings are directionally consistent with earlier trials [3, 4, 8, 12], the benefit appears attenuated, likely reflecting our broader study population, of which only 8% had high-risk features defined in prior studies. The previous trials focusing on younger female patients (≤ 50 years) with gastrointestinal cancers reported significant improvements in CR [3] and TP [8], supporting olanzapine’s greater efficacy in high-risk subgroups.

An important question is which endpoint best reflects meaningful clinical benefit in routine practice. While CR and TC are stringent efficacy markers, TP may better capture patient-centered outcomes, as even mild nausea can disrupt daily functioning. This interpretation is reinforced by the strong patient preference for continuing the OLN5 regimen.

Carboplatin at doses of AUC > 5 is classified at the higher end of MEC by ESMO/MASCC guidelines but reclassified as HEC in NCCN guidelines. Accordingly, triplet antiemetic therapy is generally recommended for patients receiving carboplatin AUC > 5, consistent with HEC protocols. Prior evidence on olanzapine in this setting has been mixed, with randomized studies reporting variable results [9–11]. In our trial, patients in the carboplatin subgroup receiving OLN5 achieved significantly higher TC rates, with numerically greater CR and TP rates, supporting guideline recommendations for adding olanzapine to standard doublet prophylaxis in high-dose carboplatin regimens. A cost-effectiveness analysis was conducted to inform broader clinical application. The additional cost of olanzapine was 98.15 THB per regimen (24.54 THB per tablet × 4 tablets). The analysis estimated 363.59–692.23 THB per additional favorable case. This modest cost may be justified given the substantial impact of CINV on quality of life. At present, olanzapine is listed in Thailand’s National List of Essential Medicines only for HEC-related CINV. We propose extending coverage to MEC, ensuring that patients can access olanzapine at no cost in this setting.

A major strength of this study is its robust randomized controlled design with a broad participant base and stratified randomization by key CINV risk factors and chemotherapy type. The trial was tailored to the Thai healthcare context, where ondansetron is the only reimbursed 5-HT3 receptor antagonist and a doublet regimen remains the MEC standard. We also evaluated OLN5 comprehensively—assessing CINV severity via both CTCAE criteria and VAS nausea scores, along with patient satisfaction and preference for future antiemetic regimens. Several limitations should be noted. First, the sample size may be inadequate to detect a moderate, clinically meaningful improvement in TP rate, as the sample size calculation was based on the expectation of a substantial benefit from adding olanzapine, whereas the true treatment effect in the context of contemporary antiemetic regimens may be more modest. Second, quality-of-life data were not collected, restricting evaluation of the full patient-centered impact. Another limitation is a single-center study, which may limit generalizability. Nonetheless, patient satisfaction was assessed and indicated a strong preference and acceptability. Finally, although the study population was broadly representative, the proportion of high-risk patients and those receiving irinotecan-based regimens was small, potentially limiting applicability to these subgroups.

## Conclusion

Olanzapine 5 mg was associated with a moderate improvement in the TP rate along with a significant gain of TC and no-nausea rates among patients receiving carboplatin at AUC > 5 or oxaliplatin- or irinotecan-based chemotherapy. These findings suggest a potential role for low-dose olanzapine in MEC, warranting further validation.

## Supplementary Information

Below is the link to the electronic supplementary material.

ESM 1 (DOCX 20.8 KB)

## Data Availability

The data that support the findings of this study are available from the Faculty of Medicine Siriraj Hospital, but restrictions apply to the availability of these data, which were used under license for the current study and so are not publicly available. The data are, however, available from the authors upon reasonable request.
