# Supplementary material for: A randomized, double-blind, placebo-controlled trial of olanzapine versus placebo plus ondansetron and dexamethasone for antiemetic prophylaxis in patients receiving oxaliplatin-, irinotecan-, or carboplatin-based chemotherapy
Source: Support Care Cancer. 2026 Feb 24;34(3):238. doi: 10.1007/s00520-026-10490-8 (PMC12929264; doi:10.1007/s00520-026-10490-8)
Supplement: Supplementary file 1 — (DOCX 20.8 KB) [file 520_2026_10490_MOESM1_ESM.docx]

**Supplementary data**

**Cost-effectiveness analysis**

We conducted a cost-effectiveness analysis using clinical outcome measures from the trial, including total protection rate, total control rate, complete response rate, and no-nausea rate, combined with micro-costing techniques to estimate associated costs. Only the costs of olanzapine and rescue medications were included, as other antiemetic drugs (ondansetron and dexamethasone) were administered identically in both the olanzapine and placebo groups. Similarly, other direct medical costs (e.g., laboratory tests), and direct non-medical costs (e.g., food and travel) were excluded, due to their equal utilization across both groups. Given that there were no extra hospital visits for rescue therapy during the trial, only additional administration costs of rescue medicines (34.29 THB per one intravenous administration) (1,2) were considered in this analysis. Costs for rescue medications were calculated based on the proportion of participants requiring rescue therapy in each group (4.3% in placebo and 1.4% in the olanzapine group). The cost of placebo was assumed to be zero, reflecting real-world clinical practice where placebo is not prescribed. Medication costs were derived from median reference prices provided by the Drug and Medical Supply Information Center (DMSIC), Ministry of Public Health, Thailand (3).

The cost-effectiveness outcomes were reported as incremental cost-effectiveness ratios (ICERs), calculated using the following formula:

$$ICER= \frac{(\mathrm{Cost}_{\mathrm{olanzapine}}-\mathrm{Cost}_{\mathrm{placebo}} )}{(\mathrm{Effect}_{\mathrm{olanzapine}} - \mathrm{Effect}_{\mathrm{placebo}})}$$

where Cost_olanzapine_ and Effect_olanzapine_ represent the total antiemetic medication cost and effects in the onlanzapine group, and Cost_placebo_ and Effect_placebo_ represent the total antiemetic medication cost and effects in the placebo group. The results of the analysis are presented in Table S1.

**Table S1** Cost-effectiveness analysis

| Antiemetic medication costs and clinical outcomes | Placebo | Olanzapine | ICERs  (THB per additional outcome) |
| --- | --- | --- | --- |
| Olanzapine 5 mg (THB per tablet) | 0 | 24.54 | NA |
| Rescue medication*  - THB per event, median (IQR)  - THB per case** | 44.83  (42.76, 101.59)  1.92 | 44.83 (NA)  0.65 | NA |
| Total antiemetic medication costs*** | 1.92 | 98.80 | NA |
| Total protection rate (%) | 55.7 | 71.0 | 633.21 THB per 1 additional case protection |
| Total control rate (%) | 38.6 | 62.3 | 407.97 THB per 1 additional controlled case |
| Complete response rate (%) | 65.7 | 79.7 | 692.23 THB per 1 additional case with complete response |
| No nausea (%) | 38.6 | 65.2 | 363.59 THB per 1 additional no-nausea case |

*Rescue medication regimens included metoclopramide 10 mg IV for one day + olanzapine 5 mg/day for 2 days, metoclopramide 10 mg IV, or ondansetron 8 mg IV. Median reference prices of metoclopramide and ondansetron were 6.40 THB per ampule and 10.54 THB per vial, respectively.

**Costs of rescue medication per case were calculated based on a proportion of participants requiring rescue therapy in each group, 4.3% in placebo and 1.4% in olanzapine groups.

*** Total antiemetic medication costs were calculated as the cost per case of rescue medication plus the cost of olanzapine, determined by multiplying the cost per tablet by four tablets per treatment case for the olanzapine group.

ICERs, incremental cost-effectiveness ratios; IQR, interquartile range; NA, not applicable; THB, Thai baht

**Reference**

1. Riewpaiboon A. Standard Cost Lists for Health Technology Assessment. [Cited September 12, 2025]. Available from: http://costingmenu.hitap.net/

2. Thailand Consumer Price Index (CPI) [Internet]. 2022 [cited September 12, 2025]. Available from: https://databank.worldbank.org/source/2?series=FP.CPI.TOTL.ZG&country=THA&savedlg=1#.

3. Drug and Medical Supply Information Center, Ministry of Public Health. [Cited September 11, 2025]. Available from: https://dmsic.moph.go.th/index/drugsearch/1
